# Foliar L-arginine and seaweed extract mitigate salinity stress in *Calendula officinalis* L. by enhancing physiological and biochemical performance

**DOI:** 10.1186/s12870-026-08566-y

**Published:** 2026-05-13

**Authors:** Pastor Zandvakili, Vahid Reza Saffari, Safoora Saadati

**Affiliations:** Department of Horticultural Science, Faculty of Agriculture, Shahid Bahonar University of Kerman, P.O. Box: 76169-133, Kerman, Iran

**Keywords:** *Calendula officinalis*, L-arginine, Seaweed extract, Salinity stress, Photosynthetic efficiency, Oxidative stress, Biostimulants

## Abstract

**Background:**

Soil salinity poses a major constraint to the productivity of salt-sensitive species such as *Calendula officinalis* L., a high-value ornamental and medicinal plant. A greenhouse study was conducted entirely under severe salinity stress (soil EC = 8.64 dS m⁻¹) and investigated the individual and combined effects of foliar-applied L-arginine (L-Arg: 0, 150, 300 mg L⁻¹) and seaweed extract (SW: 0, 1, 2 g L⁻¹) on *C. officinalis* grown under severe salinity (soil EC 8.64 dS m⁻¹) using a 3 × 3 factorial completely randomized design.

**Results:**

The co-application of 300 mg L⁻¹ L-Arg and 2 g L⁻¹ SW yielded the strongest responses, including increases in flower number by 87.5%, flower diameter by 67.0%, flower longevity by 52.0%, relative water content by 14.7%, chlorophyll a by 33.3%, chlorophyll b by 65.7%, and carotenoids by 73.9%, alongside decreases in electrolyte leakage by 34.6% and intercellular CO₂ concentration by 64.7% relative to the saline control. Main effects showed maximum increases in net photosynthetic rate (220.6% with 300 mg L⁻¹ L-Arg and 27.8% with 2 g L⁻¹ SW) and water use efficiency (279.2% with 300 mg L⁻¹ L-Arg and 71.6% with 2 g L⁻¹ SW). Catalase activity and total soluble protein rose by 98.7% and 54.9%, respectively, while root dry weight was maximized (88.2% increase) with 150 mg L⁻¹ L-Arg + 2 g L⁻¹ SW.

**Conclusion:**

The results suggest that foliar co-application of L-Arg and SW may interactively and complementarily mitigate salinity-induced osmotic, ionic, and oxidative stresses in *C. officinalis* under high saline conditions by enhancing biochemical defenses, photosynthetic performance, and morphological traits relative to the saline control, potentially offering a sustainable biostimulant strategy for cultivation in salt-affected regions.

**Supplementary Information:**

The online version contains supplementary material available at 10.1186/s12870-026-08566-y.

## Background

Soil salinity represents one of the most severe abiotic stresses, posing a significant threat to global agriculture, particularly in arid and semi-arid regions where irrigation practices exacerbate the accumulation of soluble salts in the root zone [1, 2]. Salinity stress imposes a complex triad of osmotic stress, ion toxicity, and oxidative damage on plants, disrupting critical physiological processes such as water uptake, nutrient balance, and cellular homeostasis. These effects culminate in reduced photosynthetic efficiency, impaired metabolic activities, and ultimately, diminished plant growth and yield [3–5].

*Calendula officinalis* L. (pot marigold) is a highly valued ornamental and medicinal plant. It is widely cultivated for its rich content of bioactive compounds, including carotenoids, flavonoids, and phenolics, which possess potent antioxidant, anti-inflammatory, and therapeutic properties [6, 7]. However, its cultivation is significantly hampered by moderate sensitivity to salinity, which negatively impacts both its economic and medicinal value [8, 9]. Salinity stress in *C. officinalis* manifests as stunted vegetative growth, diminished flowering, reduced chlorophyll content, and elevated oxidative stress markers such as malondialdehyde (MDA). This indicates lipid peroxidation and membrane damage under high salinity [6, 7, 10]. These challenges underscore the urgent need for effective strategies to enhance the plant’s tolerance to saline environments.

In response to salinity, plants may engage a range of defense mechanisms, such as osmotic adjustment through the accumulation of compatible solutes like proline, activation of antioxidant defense enzymes, and regulation of ion transport to support ionic homeostasis [11–14]. In *C. officinalis*, proline accumulation appears to play an important role in adaptation; however, these innate defenses can sometimes be insufficient under severe or prolonged saline conditions, which may benefit from external interventions to help maintain growth and productivity [7, 9].

Among promising biostimulants, L-arginine (L-Arg), a semi-essential amino acid, has emerged as an effective agent to alleviate salinity stress. Characterized by its guanidinium group, L-Arg serves as a rich nitrogen source and a crucial precursor for several vital metabolites. It is the biosynthetic precursor of polyamines, which stabilize membranes and nucleic acids, regulate ion channels, and enhance antioxidant enzyme activities, thereby protecting plants from salt-induced oxidative damage [15]. Additionally, L-Arg is a primary substrate for nitric oxide (NO) production via enzymatic pathways, including nitric oxide synthase (NOS)-like activity [16, 17]. Nitric oxide acts as a pivotal signaling molecule that regulates stomatal closure, activates antioxidant defenses, modulates gene expression, and regulates ion channels to maintain cellular ion homeostasis under salinity stress [18]. Furthermore, L-Arg metabolism contributes to proline biosynthesis, further aiding osmotic adjustment [19, 20]. Exogenous application of L-arginine has been consistently shown to enhance antioxidant defenses, improve nutrient uptake, and promote growth in various salt-stressed crops [21–25].

Similarly, seaweed extracts (SW), primarily derived from brown algae species like *Ascophyllum nodosum* and *Sargassum* spp., are complex mixtures containing phytohormones, polysaccharides, betaines, vitamins, minerals, and phenolic antioxidants [26, 27]. These bioactive substances collectively stimulate plant growth by promoting cell division and elongation, enhancing root and shoot development, and improving nutrient uptake efficiency [28, 29]. The polysaccharides in SWs act as elicitors that activate systemic resistance [30, 31] and enhance antioxidant enzyme activities, reducing the accumulation of reactive oxygen species (ROS) and protecting cellular structures under salt stress [32, 33]. The application of seaweed extracts has been reported to increase chlorophyll content, flower diameter, biomass, and antioxidant capacity in marigold and other crops under saline conditions [7, 8, 34]. The complementary modes of action of L-arginine and seaweed extracts offer a promising integrated approach to enhance marigold’s growth and physiological resilience under salinity stress [24]. Arginine’s role in nitrogen metabolism, polyamine and NO synthesis, and osmotic adjustment [19, 35, 36] is combined with seaweed extracts’ supply of hormones, elicitors, antioxidants, and nutrients [37–39].

Accordingly, the present study was designed to evaluate the individual and interactive effects of exogenous L-Arg and SW on the growth performance, physiological parameters, and biochemical responses of *C. officinalis* under saline soil conditions. To our knowledge, this represents the first factorial trial assessing the combined application of L-Arg and SW for salinity stress mitigation specifically in Calendula officinalis, building on similar agronomic observations in other crops while highlighting species-specific responses. The findings from this research will provide valuable insights into sustainable cultivation practices for *C. officinalis* in salt-affected environments, promoting improved productivity and quality.

## Materials and methods

### Plant material and experimental setup

The experiment was conducted in a greenhouse at Shahid Bahonar University of Kerman, Iran (30°00′N, 57°06′E; 1,754 m above sea level) from September to December 2024. Environmental conditions were maintained at a photosynthetic photon flux density (PPFD) of 600–800 µmol m⁻² s⁻¹, a 16-h photoperiod, day/night temperatures of 20.5 ± 1.5 °C / 15.0 ± 1.0 °C, and relative humidity of 20–25%. Pots (25.5 cm diameter × 25 cm height) were filled with a 2:1:1 (v/v/v) mixture of sand: clay: farmyard manure as follows: (electrical conductivity, EC = 8.64 dS m⁻¹; this salinity occurred naturally from inherent soluble salts in the locally sourced clay and farmyard manure from the saline Kerman region, without addition of NaCl or other salts; EC was measured in a 1:2 soil: water extract using a calibrated conductivity meter after thorough mixing and equilibration for 24 h). Soil salinity stability was monitored biweekly throughout the experiment by sampling soil from three randomly selected pots per treatment replicate and measuring EC using the same 1:2 soil: water extract method; values remained consistently within 8.2–9.0 dS m⁻¹, confirming no significant fluctuations due to leaching, evaporation, or other factors.

This salinity level was chosen to simulate high salt stress typical of the high-salinity soils in the semi-arid Kerman region, Iran, and to provide a stringent model for evaluating the ameliorative effects of L-arginine (L-Arg) and seaweed extract (SW) on salinity-induced physiological and biochemical impairments in *Calendula officinalis*. A non-saline control (EC < 1 dS m⁻¹) was not included in this factorial design, as the primary objective was to assess biostimulant efficacy in ameliorating high salinity stress rather than comparing stressed vs. unstressed plants. This focused approach allowed for a detailed evaluation of interactive effects under realistic saline conditions prevalent in the study region. However, this design means that improvements are expressed relative to the saline control (0 mg L⁻¹ L-Arg + 0 g L⁻¹ SW), and it is not possible to confirm whether treated plants achieved physiological performance equivalent to non-saline conditions.

Uniform 4-week-old seedlings were transplanted (one per pot). To establish a uniform nutritional baseline and eliminate nutritional variability as a confounding factor, all plants received a single, foundational application of a nutrient solution (10% N, 8% P₂O₅, 4% K₂O, 0.1% Fe-EDTA, 0.1% ZnSO₄) one week after transplanting. Throughout the experiment, no chemical pesticides or herbicides were applied. Pest and insect pressure was negligible in the controlled greenhouse, and any minor weed growth in pots was managed through manual removal.

The experiment was conducted as a 3 × 3 factorial in a completely randomized design, testing L-Arg at 0, 150, and 300 mg L⁻¹ and SW at 0, 1, and 2 g L⁻¹, with three independent replicates per treatment combination (four plants per replicate; total *n* = 108 plants). The four plants within each replicate served as subsamples; measurements from these plants were averaged to yield a single value per replicate for statistical analysis (thus, *n* = 3 true replicates per treatment, avoiding pseudoreplication). The experimental unit was each independent replicate, consisting of four pots (one plant per pot), to which treatments were applied uniformly and independently. Treatment combinations were randomly assigned to replicates using a manual randomization method, such as drawing lots. Pots were arranged randomly on greenhouse benches and rotated weekly to minimize positional effects due to micro-environmental gradients such as variations in light, temperature, or airflow across the greenhouse.

The SW was a commercial solid alkaline extract derived from the brown alga *Ascophyllum nodosum* (product: AlgeaFert Solid K^+^, manufactured by Algea AS, Kristiansund, Norway). It comprised 100% *A. nodosum* with 20% potassium (as K₂O), > 13% alginic acid, 3–7% mannitol, and 0.1% betaines, according to manufacturer specifications.

Foliar applications of L-Arg and SW were performed three times at 3-week intervals, starting at the 4–6-leaf stage. Solutions were prepared in deionized water containing 0.1% (v/v) Tween-20 as a surfactant and sprayed (50 mL per plant) until runoff on both adaxial and abaxial sides of the leaves between 08:00 and 09:00 a.m. under controlled conditions (PPFD 400–600 µmol m⁻² s⁻¹, 20–22 °C, 20–25% RH). Saline control plants received deionized water with 0.1% Tween-20. The plants were arranged in a completely randomized design, with treatments assigned randomly to replicates.

### Morphological trait assessment

#### Flower traits assessment

Flower number per plant was determined by daily counting of all newly opened buds, with the cumulative total recorded at the end of the experiment. Flower diameter was measured on three fully opened, randomly selected flowers per plant using a digital caliper (± 0.01 mm), and the mean value was calculated for each replicate. Flower longevity (days) was recorded as the time from petal opening to wilting or abscission for 10–12 tagged buds per replicate.

#### Determination of fresh and dry biomass

At final harvest, plants were uprooted, separated into shoots and roots, and their fresh weights (FW) were recorded. Samples were then oven-dried at 70 °C to a constant weight (minimum 72 h) to determine dry weight (DW). The dry matter percentage for both roots and shoots were subsequently calculated as (DW / FW) × 100 [40].

### Physiological traits

#### Photosynthetic gas exchange measurements

Photosynthetic Gas Exchange Measurements: Net photosynthetic rate (Pn), stomatal conductance (gs), intercellular CO₂ concentration (Ci), and transpiration rate (E) were quantified on fully expanded, sun-exposed leaves using a portable infrared gas analyzer (Model KR-8700, Korea Tech). Measurements were conducted under controlled settings of 1,500 µmol m⁻² s⁻¹ PAR, 400 µmol mol^− 1^ CO₂, and 25 °C leaf temperature. Water use efficiency (WUE) was calculated as Pn/E. All measurements were taken between 09:00 and 12:00 h [41].

#### Chlorophyll fluorescence analysis

Chlorophyll fluorescence parameters, including minimum fluorescence (F_0_), maximum fluorescence (F_m_), variable fluorescence (F_v_), the ratio of variable to maximum fluorescence (F_v_/F_m_), and non-photochemical quenching (NPQ), were evaluated using a pulse amplitude modulated (PAM) fluorometer (Junior-PAM, WALZ). Leaves were dark-adapted for 30 min prior to measurement [42].

### Electrolyte Leakage (EL) assay

To assess cell membrane stability, electrolyte leakage (EL) was measured according to the method of Blum and Ebercon [43]. Briefly, 0.5 g of fresh leaf discs were rinsed, immersed in 10 mL deionized water, and shaken for 24 h at 25 °C. The initial electrical conductivity (EC₁) was measured, and samples were then autoclaved at 121 °C for 20 min. After cooling, the final conductivity (EC₂) was recorded. EL was calculated as (EC₁/EC₂) × 100.

### Relative Water Content (RWC)

Leaf RWC was determined as outlined by Barrs and Weatherley [44]. RWC was calculated using the formula: RWC = [(F_W_ - D_W_) / (T_W_ - D_W_)] × 100, where FW is fresh weight, TW is turgid weight (after 4 h in distilled water), and DW is dry weight (after oven-drying at 70 °C).

### Biochemical traits

#### Chlorophyll and carotenoid quantification

Leaf pigments were extracted in 70% acetone and centrifuged at 3,500 × g for 15 min. The absorbance of the supernatant was measured at 663.2 nm, 646.8 nm, and 470 nm. The concentrations of chlorophyll a, chlorophyll b, and total carotenoids were calculated using the formulae of Lichtenthaler [45].

#### Enzyme extraction and activity assays

Fresh leaf tissue (0.5 g) was homogenized in 5 mL of 50 mM potassium phosphate buffer (pH 7.0) and centrifuged at 12,000 × g for 15 min at 4 °C. The supernatant was used as the crude enzyme extract. Catalase (CAT) activity was assayed by monitoring the decomposition of H₂O₂ at 240 nm. Peroxidase (POD) activity was quantified by measuring the oxidation of guaiacol at 470 nm [46, 47].

#### Total soluble protein assessment

Protein concentration in the enzyme extracts was determined using the Bradford assay with bovine serum albumin (BSA) as the standard [48].

#### Proline content

Free proline in leaves was determined spectrophotometrically [49]. Fresh leaf samples (0.5 g) were homogenized in 10mL 3% sulfosalicylic acid, centrifuged at 3,500×g, and 2mL supernatant reacted with 2mL acid ninhydrin and 2mL glacial acetic acid, incubated at 100 °C for 1 h. After cooling, 4mL toluene was added, the mixture vortexed, and absorbance of the upper (toluene) phase read at 520 nm. Proline content was calculated using a standard curve of L-proline.

### Statistical analysis

Data were analyzed by two-way ANOVA using SAS software (ver. 9.4). Prior to analysis, normality (Shapiro–Wilk test) and homoscedasticity (Levene’s test) were confirmed for all variables; no transformations were required. Main effects of L-Arg, SW, and their interaction were evaluated. Means were separated by Duncan’s multiple range test at *p* ≤ 0.05. Significance is indicated as: ns (not significant), * (*p* ≤ 0.05), ** (*p* ≤ 0.01), *** (*p* ≤ 0.001). All data are presented as mean ± standard error (SE).

## Results

The application of L-arginine (L-Arg) and seaweed extract (SW) significantly mitigated the adverse effects of salinity on C. officinalis, with pronounced interactive effects observed across multiple traits (Tables 1 and 2, and Supplementary Tables S1 and S2 for treatment means ± SE).

**Table 1:** Two-way ANOVA results showing the effects of L-arginine (L-Arg) and Seaweed (SW) treatments on selected physiological traits and photosynthetic parameters of pot marigold (*Calendula officinalis* L.)

SOV	df	F value											
		FN	FD	FL	RDM	SDM	EL	RWC	Pn	E	gs	Ci	WUE
L-Arg	2	26.26 ^***^	174.19 ^***^	24.12 ^***^	1.52 ^ns^	14.32 ^***^	16.38 ^***^	13.24 ^***^	313.55 ^***^	7.35 ^**^	41.82 ^***^	337.41 ^***^	112.74 ^***^
SW	2	25.60 ^***^	29.24 ^***^	42.68 ^***^	31.46 ^***^	27.41 ^***^	105.49 ^***^	7.50 ^**^	15.45 ^***^	18.71 ^***^	51.53 ^***^	3.38 ^ns^	22.81 ^***^
L-Arg × SW	4	3.83 ^*^	5.28 ^**^	3.50 ^*^	19.53 ^***^	0.23 ^ns^	3.10 ^*^	6.23 ^**^	1.12 ^ns^	0.69 ^ns^	2.03 ^ns^	3.05 ^*^	1.80 ^ns^
Error	18												
Cv (%)		7.4	3.8	5.3	8.5	4.1	4.3	2.6	9.5	10.3	7.4	7.1	17.4

Statistical significance is indicated by asterisks (*p ≤ 0.05, **p ≤ 0.01, and ***p ≤ 0.001), while ‘ns’ indicates non-significance

*FN* Flower number, *FD* Flower diameter, *FL* Flower longevity, *RDM* Root dry matter, *SDM* Shoot dry matter, *EL* Electrolyte leakage, *RWC* Relative water content, *Pn* Net photosynthetic rate, *E* Transpiration rate, *gs* Stomatal conductance, *Ci* Intercellular CO₂ concentration, *WUE* Water use efficiency

**Table 2:** Two-way ANOVA results showing the effects of L-arginine (L-Arg) and Seaweed (SW) on chlorophyll fluorescence characteristics, photosynthetic pigment contents and biochemical parameters of pot marigold (*Calendula officinalis* L.)

SOV	df	F value													
		F_0_	F_v_	F_m_	F_v_/F_m_	NPQ	Chl a	Chl b	Chl T	Car	CAT	POD	TSP	PC	
L-Arg	2	30.69 ^***^	29.87 ^***^	28.73 ^***^	27.79 ^***^	145.38 ^***^	55.95 ^***^	13.69 ^***^	8.17 ^**^	191.45 ^***^	128.26 ^***^	93.47 ^***^	64.67 ^***^	9.04 ^**^	
SW	2	85.87 ^***^	8.61^**^	2.31 ^ns^	37.25 ^***^	135.86 ^***^	51.98 ^***^	50.21 ^***^	8.45 ^**^	152.18 ^***^	40.32 ^***^	47.72 ^***^	21.43 ^***^	2.61 ^ns^	
L-Arg × SW	4	6.01 ^**^	3.91 ^*^	3.23 ^*^	4.60 ^**^	1.63 ^ns^	6.48 ^**^	4.49 ^*^	16.30 ^***^	4.10 ^*^	3.95 ^*^	0.99 ^ns^	12.20 ^***^	0.10 ^ns^	
Error	18														
Cv (%)		2.1	4.4	3.2	1.6	6.6	2.7	6. 9	5.3	2.8	6.4	10.4	5.2	9.5	

Statistical significance is indicated by asterisks (*p ≤ 0.05, **p ≤ 0.01, and ***p ≤ 0.001), while ‘ns’ indicates non-significance

*F*_0_ Minimum fluorescence, *F*_v_ Variable fluorescence, *F*_m_ Maximum fluorescence, *F*_v_/*F*_m_ Maximum quantum yield of PSII, *NPQ* Non-photochemical quenching, *Chl a* Chlorophyll a, *Chl b* Chlorophyll b, *Chl T* Total chlorophyll, *Car* Total carotenoids, *CAT* Catalase activity, *POD* Peroxidase activity, *TSP* Total soluble protein, *PC* Proline content

### Morphological traits

**Fig. 1:**
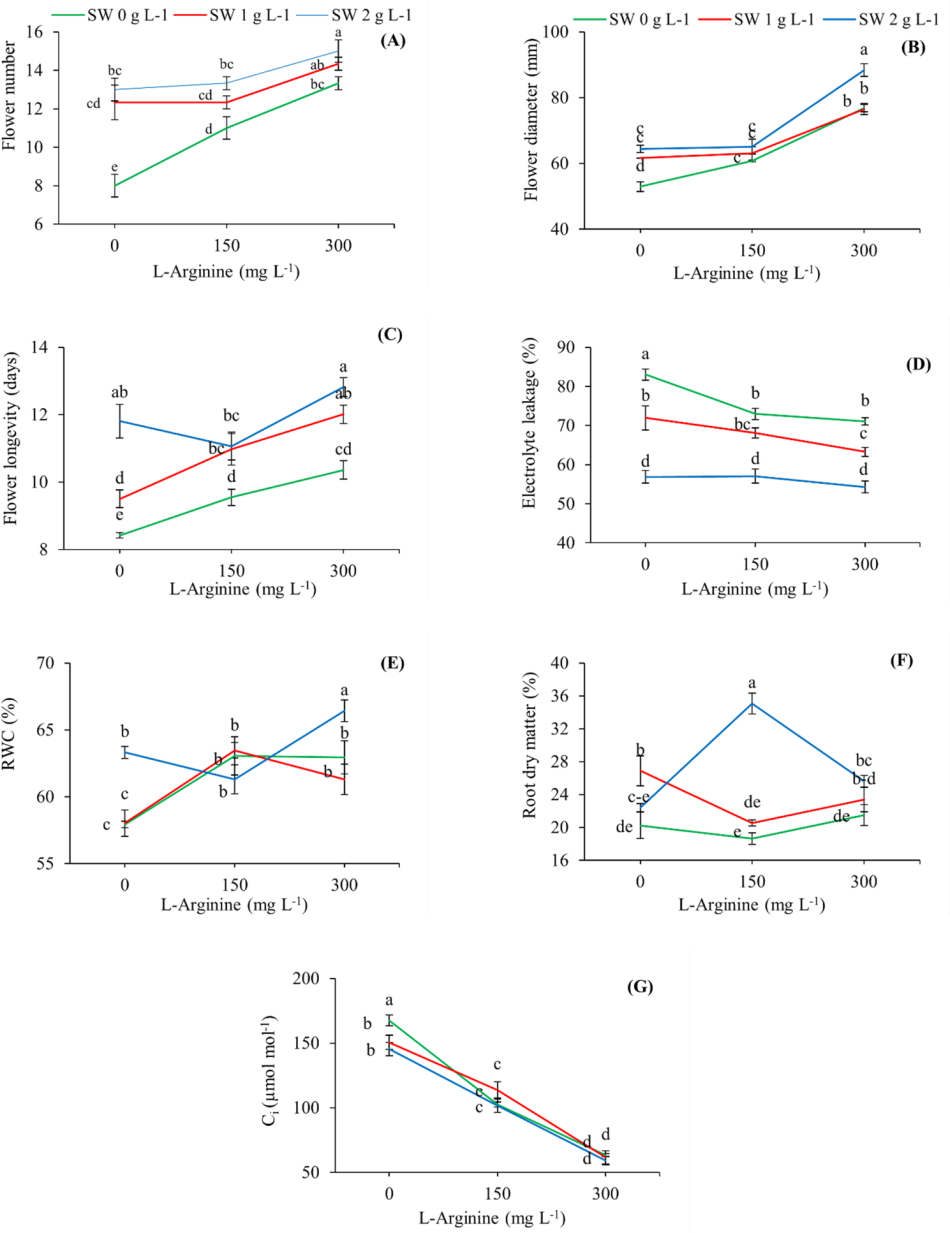
Interaction effects of L-arginine (L-Arg) and seaweed extract (SW) on pot marigold (*Calendula officinalis* L.) grown entirely under saline conditions: flower number (**A**), flower diameter (**B**), flower longevity (**C**), electrolyte leakage (**D**), relative water content (RWC; **E**), root dry matter (**F**), intercellular CO₂ concentration (Ci; **G**). Means ± SE (*n* = 3). Different letters denote statistically significant differences based on Duncan’s Multiple Range Test at the 5% probability level (*p* ≤ 0.05). Saline control: 0 mg L⁻¹ L-Arg + 0 g L⁻¹ SW

A significant interaction between L-Arg and SW was observed for key floral characteristics. The combined application of 300 mg L⁻¹ L-Arg and 2 g L⁻¹ SW produced the most pronounced improvements, increasing flower number to 15.00 per plant (an 87.5% improvement over the saline control’s 8.00; F = 3.83, *p* ≤ 0.05; Fig. 1A; Table 1 and Supplementary Table S1). This treatment also enhanced flower diameter to 88.35 mm (67.0% greater than the saline control’s 52.91 mm; F = 5.28, *p* ≤ 0.01; Fig. 1B; Table 1 and Supplementary Table S1) and flower longevity to 12.81 days (52.0% higher than the saline control’s 8.42 days; F = 3.50, *p* ≤ 0.05; Fig. 1C; Table 1 and Supplementary Table S1).

**Fig. 2:**
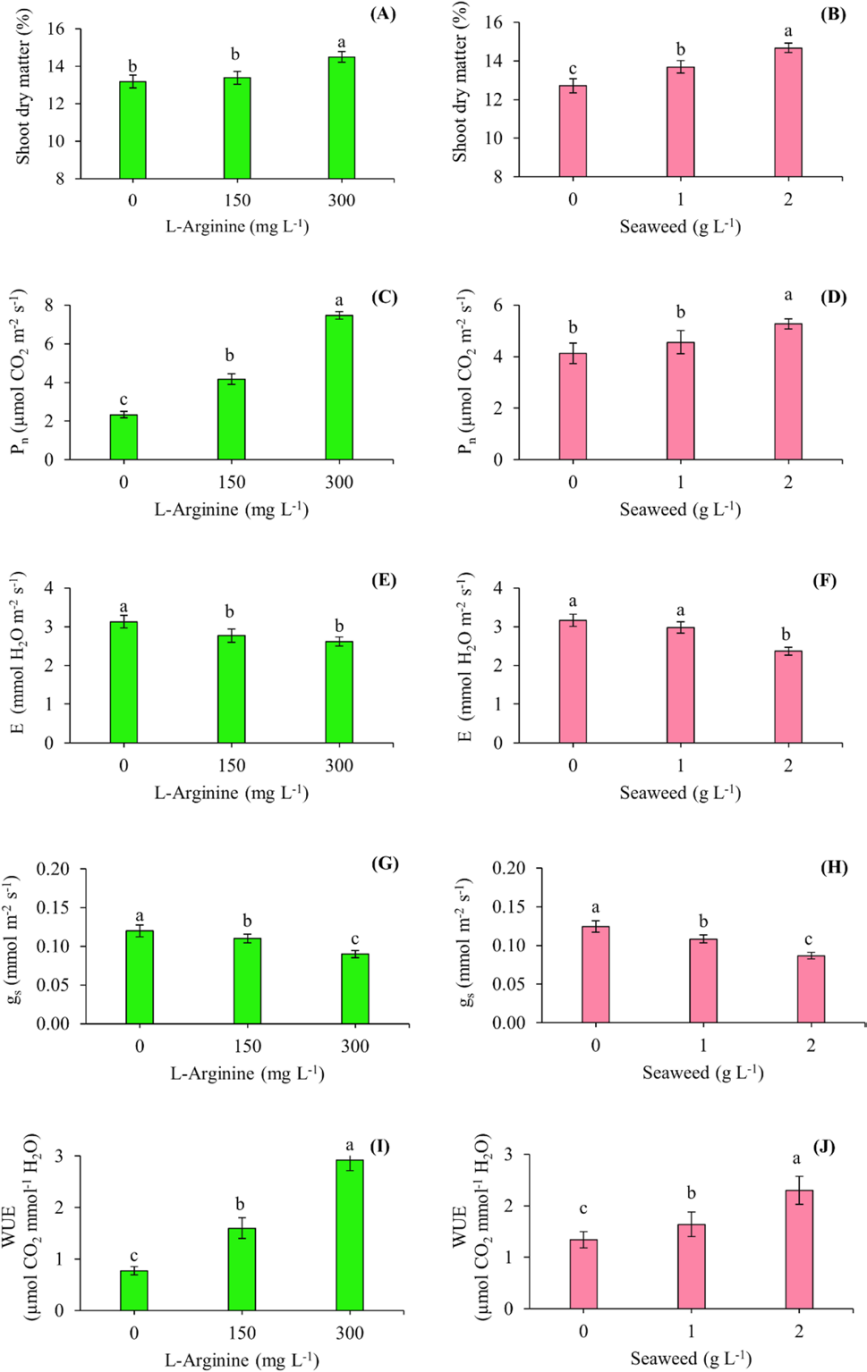
Simple effects of L-arginine (L-Arg; **A**, **C**, **E**, **G**) and seaweed extract (SW; **B**, **D**, **F**, **H**) on pot marigold (*Calendula officinalis* L.) grown entirely under saline conditions: shoot dry matter (**A**, **B**), photosynthesis rate (P_n_; **C**, **D**), transpiration rate (E; **E**, **F**), stomatal conductance (g_s_; **G**, **H**), and water use efficiency (WUE; **I**, **J**). Means ± SE (*n* = 3). Different letters above bars denote statistically significant differences based on Duncan’s Multiple Range Test at the 5% probability level (*p* ≤ 0.05). Saline control: 0 mg L⁻¹ L-Arg + 0 g L⁻¹ SW

For biomass allocation, a significant interaction was also found for root dry matter. The combination of 150 mg L⁻¹ L-Arg with 2 g L⁻¹ SW was most effective, maximizing root dry matter at 35.08% (an 88.2% increase over the lowest value of 18.63%; F = 19.53, *p* ≤ 0.001; Fig. 1F; Table 1 and Supplementary Table S1). No significant interaction was found for shoot dry matter percentage (F = 0.23, *p* > 0.05, Table 1 and Supplementary Table S1); however, significant main effects were observed. Application of 2 g L⁻¹ SW increased shoot dry matter to 14.67% (15.4% above the 12.71% saline control), and 300 mg L⁻¹ L-Arg raised it to 14.50% (10.0% higher than the 13.18% saline control) (Figs. 2A, B).

### Physiological traits and gas exchange

The optimal treatment (300 mg L⁻¹ L-Arg + 2 g L⁻¹ SW) significantly improved plant water status and membrane stability. This combination reduced electrolyte leakage to 54.30% (a 34.6% decrease from the saline control’s 83.01%; F = 3.10, *p* ≤ 0.05; Fig. 1D; Table 1 and Supplementary Table S1) and increased relative water content to 66.43% (a 14.7% rise over the saline control’s 57.92%; F = 6.23, *p* ≤ 0.01; Fig. 1E; Table 1 and Supplementary Table S1).

In terms of gas exchange, no significant interaction was observed for net photosynthetic rate (Pn), transpiration rate (E), stomatal conductance (gs), or water use efficiency (WUE) (F = 1.12, 0.69, 2.03, 1.80 respectively, *p* > 0.05, Table 1 and Supplementary Table S1). However, both biostimulants exerted significant main effects.

Foliar application of 300 mg L⁻¹ L-Arg increased Pn to 7.47 µmol m⁻² s⁻¹ (a 220.6% rise over the 2.33 µmol m⁻² s⁻¹ in the 0 mg L⁻¹ L-Arg treatment), while 2 g L⁻¹ SW raised Pn to 5.28 µmol m⁻² s⁻¹ (a 27.8% increase over the 4.13 µmol m⁻² s⁻¹ in the 0 g L⁻¹ SW treatment) (Figs. 2C, D, and Supplementary Table S1 for means ± SE). Conversely, both treatments reduced E and gs. L-Arg (300 mg L⁻¹) decreased E to 2.62 mmol⁻¹ H₂O m⁻² s⁻¹ and gs to 0.09 mmol m⁻² s⁻¹, while SW (2 g L⁻¹) reduced E to 2.36 mmol⁻¹ H₂O m⁻² s⁻¹ and gs to 0.09 mmol m⁻² s⁻¹ (Figs. 2E–G).

Both L-Arg and SW extract independently enhanced WUE under saline conditions. Foliar application of 300 mg L⁻¹ L-Arg increased WUE to 2.92 µmol CO₂ mmol⁻¹ H₂O (a 279.2% increase over the 0.77 in the 0 mg L⁻¹ L-Arg treatment), while 2 g L⁻¹ SW raised WUE to 2.30 µmol CO₂ mmol⁻¹ H₂O (a 71.6% increase over the 1.34 in the 0 g L⁻¹ SW treatment) (Figs. 2I, J; Table 1). Crucially, a significant interaction was found for intercellular CO₂ concentration (C_i_). The 300 mg L⁻¹ L-Arg + 2 g L⁻¹ SW treatment reduced C_i_ to 59.12 µmol mol^− 1^ (a 64.7% decrease from the saline control’s 167.42 µmol mol^− 1^; F = 3.05, *p* ≤ 0.01; Fig. 1G; Table 1 and Supplementary Table S1).

### Photosynthetic apparatus: fluorescence and pigments

**Fig. 3:**
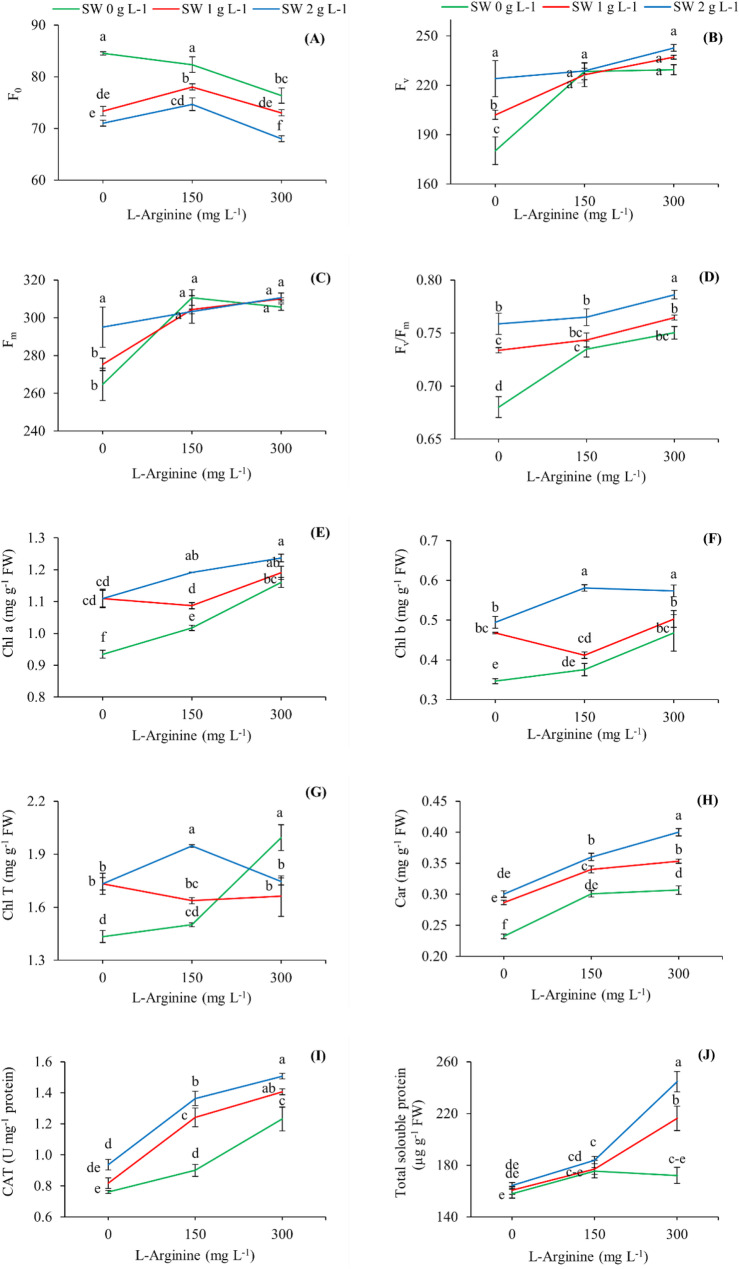
Interaction effects of L-arginine (L-Arg) and seaweed extract (SW) on pot marigold (*Calendula officinalis* L.) grown entirely under saline conditions: minimum fluorescence (F_0_; **A**), variable fluorescence (F_v_; **B**), maximum fluorescence (F_m_; **C**), maximum quantum yield of photosystem II (F_v_/F_m_; **D**), chlorophyll a content (**E**), chlorophyll b content (**F**), total chlorophyll content (**G**), total carotenoid content (**H**), catalase activity (CAT; **I**), and total soluble protein content (**j**). Means ± SE (*n* = 3). Different letters denote statistically significant differences based on Duncan’s Multiple Range Test at the 5% probability level (*p* ≤ 0.05). Saline control: 0 mg L⁻¹ L-Arg + 0 g L⁻¹ SW

**Fig. 4:**
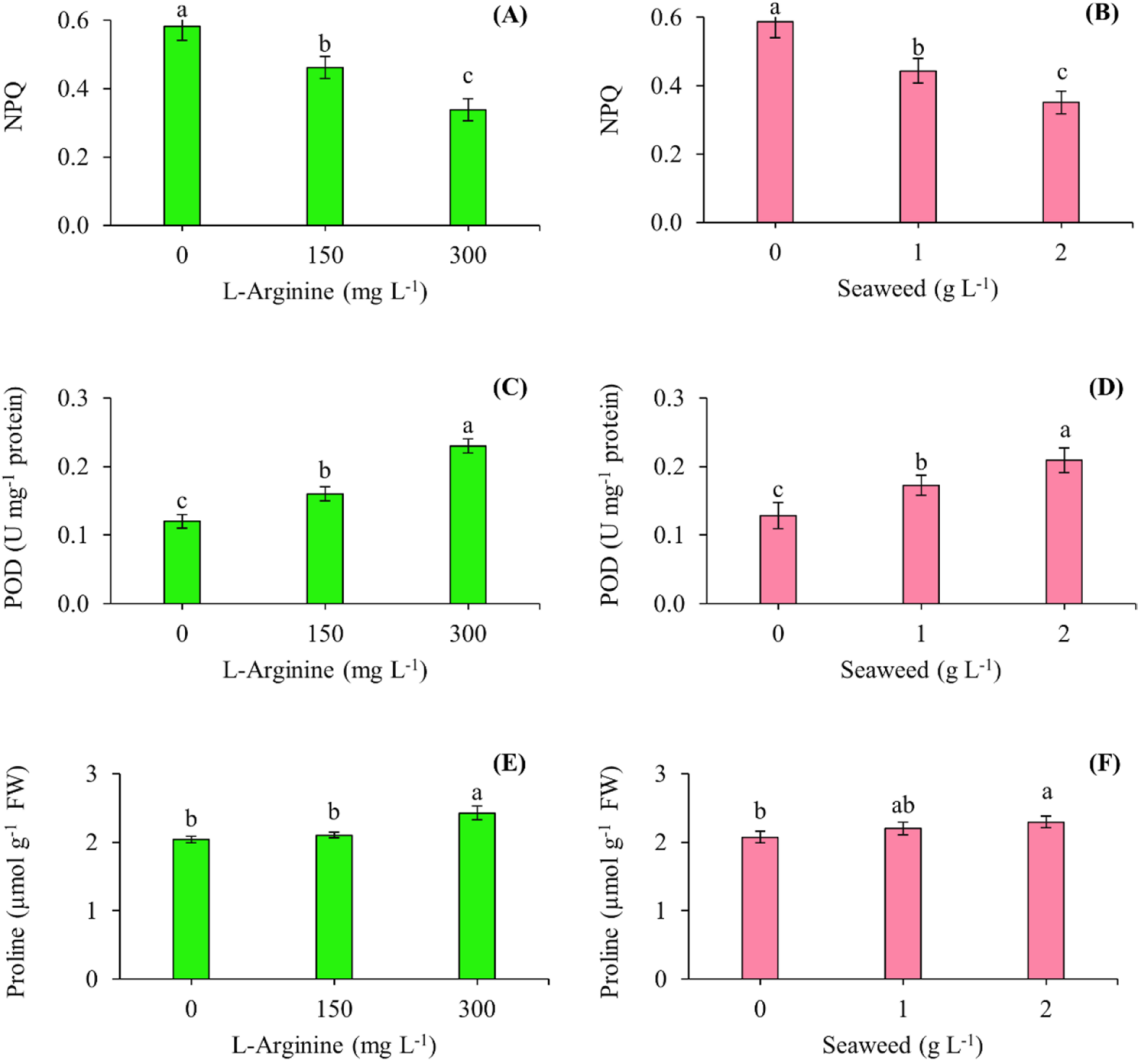
Simple effects of L-arginine (L-Arg; **a**, **c**, **e**) and seaweed extract (SW; **b**, **d**, **f**) on pot marigold (*Calendula officinalis* L.) grown entirely under saline conditions: non-photochemical quenching (NPQ; **A**, **B**), peroxidase activity (POD; **C**, **D**), and proline content (**E**, **F**). Means ± SE (*n* = 3). Different letters above bars denote statistically significant differences based on Duncan’s Multiple Range Test at the 5% probability level (*p* ≤ 0.05). Saline control: 0 mg L⁻¹ L-Arg + 0 g L⁻¹ SW

Under the 300 mg L⁻¹ L-Arg + 2 g L⁻¹ SW treatment, minimum fluorescence (F_0_) decreased to 68.00 (19.5% below the saline control’s 84.50; F = 6.01, *p* ≤ 0.01; Fig. 3A; Table 1 and Supplementary Table S2), while variable fluorescence (F_v_) and maximum fluorescence (F_m_) increased to 242.67 and 310.67, respectively (34.7% and 17.4% higher than the saline control; F = 3.91–3.23, *p* ≤ 0.05; Figs. 3B, C; Table 1 and Supplementary Table S2). Consequently, the maximum quantum yield (F_v_/F_m_) rose to 0.79 (a 16.2% increase over the saline control’s 0.68; F = 4.60, *p* ≤ 0.01; Fig. 3D; Table 1 and Supplementary Table S2). For non-photochemical quenching (NPQ), no interaction was found (F = 1.63, *p* > 0.05, Table 1 and Supplementary Table S2), but main effects showed both 300 mg L⁻¹ L-Arg and 2 g L⁻¹ SW reduced NPQ by approximately 41% (Figs. 4A, B).

Interactive effects showed chlorophyll a increased to 1.24 mg g⁻¹ fresh weight (33.3% above the saline control’s 0.93) with 300 mg L⁻¹ L-Arg + 2 g L⁻¹ SW (F = 6.48, *p* ≤ 0.01; Fig. 3E; Table 1 and Supplementary Table S2). Chlorophyll b levels were elevated by both 150 mg L⁻¹ and 300 mg L⁻¹ L-Arg when combined with 2 g L⁻¹ SW, with the highest value of 0.58 mg g⁻¹ fresh weight (65.7% higher than the saline control’s 0.35) (F = 4.49, *p* ≤ 0.05; Fig. 3F; Table 1 and Supplementary Table S2). Total chlorophyll reached 1.99 mg g⁻¹ fresh weight, 39.2% above the saline control’s 1.43 (F = 16.30, *p* ≤ 0.001; Fig. 3G; Table 1 and Supplementary Table S2) at 300 mg L⁻¹ L-Arg + 0 g L⁻¹ SW, and total carotenoids hit 0.40 mg g⁻¹ fresh weight, 73.9% higher than the saline control’s 0.23 (F = 4.10, *p* ≤ 0.05; Fig. 3H; Table 1 and Supplementary Table S2) at 300 mg L⁻¹ L-Arg + 2 g L⁻¹ SW.

### Biochemical defenses

The interactive effect of the treatments was further evident in the biochemical responses. The 300 mg L⁻¹ L-Arg + 2 g L⁻¹ SW combination led to the most substantial increases in catalase (CAT) activity to 1.51 U mg⁻¹ protein (98.7% above the saline control’s 0.76; F = 3.95, *p* ≤ 0.05; Fig. 3I; Table 1 and Supplementary Table S2) and total soluble protein (TSP) to 244.60 mg g⁻¹ fresh weight (54.9% higher than the saline control’s 157.81; F = 12.20, *p* ≤ 0.001; Fig. 3J; Table 1 and Supplementary Table S2).

For other biochemical parameters, significant main effects were observed but no interaction. Peroxidase (POD) activity increased by 91.7% with 300 mg L⁻¹ L-Arg and by 61.5% with 2 g L⁻¹ SW (Figs. 4C, D; Table 1 and Supplementary Table S2). Similarly, proline content rose by 19.1% with 300 mg L⁻¹ L-Arg and by 11.1% with 2 g L⁻¹ SW (Figs. 4E, F; Table 1 and Supplementary Table S2).

## Discussion

This study highlights the interactive and complementary efficacy of L-arginine (L-Arg) and seaweed extract (SW) in mitigating salinity stress in *C. officinalis* by addressing physiological, biochemical, and morphological impairments caused by high salt levels. The combined treatment, particularly 300 mg L⁻¹ L-Arg + 2 g L⁻¹ SW, orchestrated a robust defense response, alleviating osmotic, ionic, and oxidative stresses, relatively improving plant growth and productivity under high saline conditions. This integrated approach outperformed individual applications, underscoring the potential of combined biostimulants as a sustainable strategy for saline agriculture. While the novelty of this work lies primarily in its agronomic application as the first factorial L-Arg + SW salinity trial in *C. officinalis*, the mechanistic insights discussed herein are hypothesized based on our data and prior literature, rather than being directly validated through targeted molecular assays.

### Interactive effects on growth and resource allocation

The combined L-Arg and SW treatment markedly improved reproductive output and root development, as evidenced by significant increases in flower number, diameter, and longevity relative to the saline control. These results suggest that the combined action of the treatments not only countered salinity stress but also redirected resources toward reproductive organs. L-Arg contributes by serving as a precursor for polyamines (e.g., spermidine, spermine) and nitric oxide (NO). Polyamines are polycationic molecules. They bind to negatively charged phospholipids, which stabilizes cellular and organellar membranes. They also regulate ion channels to maintain K⁺/Na⁺ homeostasis under salinity stress [50]. Additionally, polyamines inhibit membrane-bound phospholipases and proteases. This reduces lipid peroxidation and protein degradation [51]. These observations are consistent with previous reports on L-Arg that suggest it may enhance inflorescence numbers in *C. officinalis* [24] and leaf numbers in *Helianthus annuus* under salinity [52], as well as enhanced flower yield in *Tagetes erecta* [53] and reproductive development in *Triticum aestivum* under drought [54]. Complementing L-Arg, SW serves as an external biostimulant, rich in cytokinins that delay leaf and flower senescence, directly contributing to prolonged flower longevity [55]. Auxins in SW promote lateral root initiation, explaining the observed increase in root dry matter [37]. Notably, the intermediate dose of L-Arg (150 mg L⁻¹) combined with high SW (2 g L⁻¹) maximized root dry matter, suggesting a hormetic response where moderate L-Arg levels optimize polyamine and NO signaling without inducing potential toxicity or metabolic overload at higher concentrations [19, 35]. Moreover, SW provides osmoprotectants like betaines (e.g., glycine betaine), which complement L-Arg-derived proline to shield cellular structures and enzymes from osmotic damage [56]. These osmoprotectants stabilize protein folding and enzyme activity. They do so by excluding water from hydration spheres, which preserves macromolecular integrity under low water potential [57]. Consistent with these results, SW has been shown to enhance flower yield in *Callistephus chinensis* [58] and improve floral traits in *Echium amoenum* [59] and *Amaranthus tricolor* [60] under salinity. Recent studies also report extended flower longevity in *Dahlia variabilis* [61] and improved flower quality in *Lavandula officinalis* under salt stress [62], reinforcing our findings. For context, under non-saline or optimal conditions, *C. officinalis* typically produces > 4–44 flowers per plant (with regular deadheading) and flower diameters of 45–75 mm [8, 62–64]. In our saline control (~ 8 flowers/plant, ~ 53 mm diameter), the optimal treatment (300 mg L⁻¹ L-Arg + 2 g L⁻¹ SW) increased these to ~ 15 flowers/plant and ~ 88 mm—substantially alleviating salinity damage and approaching non-stressed benchmarks based on literature reports (without direct comparison in this study). These findings suggest that this biostimulant approach may be transferable to other ornamental species facing similar salinity challenges, though further validation in diverse crops is warranted.

### Restoration of physiological homeostasis and water use efficiency

The combined application of L-Arg and SW effectively alleviated salinity-induced disruptions in water relations and membrane integrity in *Calendula officinalis*. The significant reduction in electrolyte leakage (EL)and concurrent increase in relative water content (RWC) reflect relatively improved cellular stability and enhanced turgor maintenance relative to the saline control. Mechanistically, L-Arg-derived polyamines likely integrate into the lipid bilayer. This reduces membrane fluidity and permeability to prevent ion leakage [65]. Polyamines also adjust the membrane’s phase transition temperature. This bolsters resilience against salt-induced phase separations [66, 67]. These findings are consistent with previous reports showing reduced EL in *Triticum aestivum* with L-Arg [21] and in *Zea mays* with *Ecklonia maxima* SW [68]. Recent research further supports this, with L-Arg improving membrane stability in *Glycine max* [69] and SW reducing lipid peroxidation in *Brassica napus* [70].

The treatments also markedly improved water use efficiency (WUE) through significant main effects, with 300 mg L⁻¹ L-Arg boosting WUE by 279.2% (from 0.77 to 2.92 µmol CO₂ mmol⁻¹ H₂O) and 2 g L⁻¹ SW increasing it by 71.6% (from 1.34 to 2.30 µmol CO₂ mmol⁻¹ H₂O), indicating substantial recovery of photosynthetic performance. While this percentage appears high, it primarily reflects the restoration of WUE from a severely depressed baseline under salinity stress toward values typical of non-stressed plants. The absolute increase (e.g., + 2.15 µmol CO₂ mmol⁻¹ H₂O for L-Arg) signifies effective mitigation of stomatal and metabolic limitations, enhancing carbon fixation per unit water transpired. Beyond decreasing stomatal conductance (gs), the combined action of L-Arg and SW may contribute to improved CO₂ utilization, as suggested by the observed patterns in gas exchange parameters (e.g., decreases in gs, E, and Ci, and increases in Pn and WUE). This larger relative change in WUE compared to the modest improvement in Fv/Fm (from 0.68 to 0.79, + 16.2%) is expected, as Fv/Fm measures only PSII photochemical efficiency, whereas WUE encompasses integrated enhancements in gas exchange, including reduced transpiration and optimized CO₂ assimilation that are disproportionately amplified from a low-stress baseline. These patterns are consistent with previous reports on enhanced photosynthetic gas exchange in *Hordeum vulgare* under salinity stress [71] and improved photochemical efficiency and water use in *Arabidopsis thaliana* [72] and *Spinacia oleracea* [73] with SW under abiotic stress, though direct enzymatic measurements (e.g., of Rubisco activity or carboxylation capacity) were not conducted in this study. (see Supplementary Table S1 for absolute values ± SE).

### Reinvigoration of the photosynthetic machinery

The combined application of L-Arg and SW significantly enhanced the photosynthetic apparatus of *Calendula officinalis* under salinity stress, as evidenced by improved chlorophyll fluorescence parameters (F_v_/F_m_, F_v_, F_m_) relative to the saline control. These improvements indicate a healthier and more efficient Photosystem II (PSII). A higher F_v_/F_m_ ratio reflects reduced photoinhibitory damage to the D1 protein in the PSII reaction center, suggesting enhanced photoprotection and repair. This effect may be associated with the enhanced antioxidant enzyme system and potentially supported by direct scavenging of ROS by SW-derived phenolic compounds, which could shield the D1 protein from oxidative degradation, thereby improving electron transport efficiency [74, 75]. Consistent with these findings, enhanced F_v_/F_m_ has been reported with L-Arg in *Zea mays* [22] and *Malus hupehensis* [76], and with SW in *Carthamus tinctorius* [77] and *Echium amoenum* [59] under saline conditions.

The treatments also significantly increased photosynthetic pigment levels relative to the saline control. Salinity typically accelerates chlorophyll degradation via chlorophyllase activity and ROS-induced damage. The L-Arg and SW combination counteracted this through multiple mechanisms: proline (from L-Arg) and betaines (from SW) stabilized thylakoid membranes, preserving pigment-protein complexes, while the bolstered antioxidant system mitigated oxidative bleaching [22, 34, 78, 79]. Furthermore, SW supplied essential micronutrients, such as Mg²⁺ and Fe²⁺, critical for chlorophyll biosynthesis and electron transport [29]. These findings align with increased pigment levels in *C. officinalis* with L-Arg [24] and SW-mediated pigment stabilization in *Echium amoenum* [59]. Under non-saline conditions, total chlorophyll content in *C. officinalis* leaves typically ranges from 1 to 4 mg g⁻¹ fresh weight [24, 63, 80, 81]. Our saline control reflected marked decline (~ 1.43 mg g⁻¹ total chlorophyll), while the best treatment raised levels toward ~ 1.99 mg g⁻¹, demonstrating effective partial mitigation based on literature reports (without direct comparison in this study).

### Bolstering the antioxidant defense system

The combined application of L-Arg and SW significantly enhanced the enzymatic antioxidant defenses of *C. officinalis* under salinity stress, as evidenced by increased CAT and POD activities. This convergence of effects establishes a robust, multi-compartmental defense strategy against oxidative stress. CAT, predominantly found in peroxisomes, efficiently breaks down hydrogen peroxide (H₂O₂) into water and oxygen, mitigating oxidative damage. Meanwhile, POD, active in the apoplast and cytosol, utilizes phenolic compounds—abundantly provided by SW—as electron donors to neutralize H₂O₂, further protecting cellular structures [82].

Additionally, L-Arg-derived polyamines contribute direct free radical scavenging, particularly against hydroxyl radicals, bolstering non-enzymatic defenses [83]. The combined action of L-Arg and SW may be associated with amplification of the Asada-Halliwell (ascorbate-glutathione) cycle, potentially enhancing the activities of key enzymes such as ascorbate peroxidase (APX), glutathione reductase (GR), monodehydroascorbate reductase (MDHAR), and dehydroascorbate reductase (DHAR), although this was not directly measured in the present study. Previous studies suggest this possibility, as they demonstrate that SW enhances APX and GR activities in rice under salinity stress [55], while polyamines upregulate the entire cycle in mung bean [84]. Such a coordinated response could create an effective network for reactive oxygen species (ROS) detoxification.

Comparable findings include enhanced antioxidant activity in *Salicornia europaea* with L-Arg application [23] and increased CAT and POD activities in *Vigna mungo* with SW treatment [85]. Recent research [86] further corroborates the role of SW in boosting antioxidant enzyme activities, aligning with our results.

### Mechanism of interactive and complementary effects

The superior performance of the combined treatment can be explained by the distinct yet complementary modes of action of L-Arg and SW. L-Arg functions primarily as an internal metabolic regulator. It modulates key signaling pathways (NO, polyamines) and precursor synthesis (proline) to enhance the plant’s innate stress tolerance mechanisms from within. In contrast, SW acts as an external biostimulatory supply, providing a rich cocktail of phytohormones, nutrients, antioxidants, and elicitors that directly support growth and activate defense pathways. Their integration creates a holistic strategy where internal signaling is amplified by external support, leading to a more resilient and robust physiological state than either treatment could achieve alone. Importantly, these mechanisms are hypothesized based on established literature, and direct mechanistic validation (e.g., through NO quantification or gene expression analysis) would enhance future studies.

### Limitations and future perspectives

While the present findings demonstrate clear benefits of L-arginine and seaweed extract under saline conditions, several limitations must be acknowledged. The absence of a non-saline control means that observed improvements reflect only the mitigation of salt-induced damage relative to the untreated saline control, rather than full restoration to unstressed performance. The experiment employed three independent biological replicates per treatment (*n* = 3), with four plants averaged per replicate—a design that is standard and statistically appropriate for resource-intensive greenhouse studies involving multiple physiological and biochemical parameters. Duncan’s multiple range test following two-way ANOVA was selected as it is widely accepted in comparable horticultural research. Additionally, the commercial seaweed extract contained 20% K₂O together with alginates, mannitol, betaines and micronutrients; consequently, some growth and physiological enhancements may partly derive from direct nutrient supply in addition to biostimulant effects. Because leaf nutrient analysis was not performed, the relative contributions of nutritional versus signaling-mediated mechanisms could not be disentangled. Finally, salinity stress severity was assessed indirectly through physiological indicators (e.g., Fv/Fm = 0.68 in the saline control, indicating moderate photoinhibition), without direct quantification of tissue ions, reactive oxygen species, or lipid peroxidation.

Future research should test specific hypotheses regarding the underlying mechanisms, with particular emphasis on nitric oxide (NO) signaling derived from L-arginine. NO is proposed to mediate post-translational modifications (e.g., S-nitrosylation) of proteins to regulate gene expression and metabolic pathways [87], potentially including activation of Rubisco via S-nitrosylation of Rubisco activase [67, 88, 89], phosphorylation-dependent enhancement of aquaporin function [90], transcriptional upregulation of psbA for PSII repair [91], and activation of NAC and WRKY transcription factors to upregulate antioxidant enzyme genes [92, 93], consistent with prior evidence linking L-arginine-derived NO to antioxidant defense [17].

Future validation requires direct NO quantification, gene expression profiling, proteomic analysis of S-nitrosylated/phosphorylated proteins, and ion/oxidative stress biomarkers. The L-arginine + seaweed extract strategy should also be tested in other ornamental and medicinal species to confirm broader applicability under salinity stress.

## Conclusion

In conclusion, this study, the first factorial trial of its kind in *C. officinalis*, provides compelling evidence that the foliar application of L-arginine and seaweed extract acts interactively and complementarily to mitigate the adverse effects of high salinity and relatively enhance the salt tolerance of *C. officinalis* under saline conditions. The combined treatment of 300 mg L⁻¹ L-arginine and 2 g L⁻¹ seaweed extract was the most effective. While the underlying mechanisms are inferred from our physiological and biochemical data rather than directly demonstrated, the results are clear. By simultaneously relatively improving morphological traits, alleviating disruptions in physiological homeostasis, relatively enhancing the photosynthetic machinery, and bolstering biochemical defenses, this combination offers a powerful, sustainable, and practical tool for cultivating *C. officinalis* in salt-affected regions. The success of this approach suggests it may be transferable to other salt-sensitive ornamental species, though this requires further investigation, contributing to strategies for addressing the global challenge of soil salinity. Nevertheless, to translate these promising greenhouse findings into practical agricultural applications and to fully elucidate the underlying molecular mechanisms, future research should include field trials under variable environmental conditions and molecular validation of the proposed pathways.

## Supplementary Information

Supplementary Material 1.

Supplementary Material 2.

## Data Availability

All data supporting the findings of this study are available within the paper and its Supplementary Information. Supplementary Tables S1 and S2 provides absolute values and variance ranges (mean ± SE) for all measured physiological, morphological, and biochemical parameters. The raw datasets generated and/or analysed during the current study are included in the Supplementary Information and are also available from the corresponding author upon reasonable request.
