# Supplementary material for: Foliar L-arginine and seaweed extract mitigate salinity stress in Calendula officinalis L. by enhancing physiological and biochemical performance
Source: BMC Plant Biol. 2026 May 13;26:1143. doi: 10.1186/s12870-026-08566-y (PMC13348960; doi:10.1186/s12870-026-08566-y)
Supplement: Supplementary file 1 — Supplementary Material 1. [file 12870_2026_8566_MOESM1_ESM.docx]

**Supplementary Table S1.** Mean values (± standard error) for physiological traits and photosynthetic parameters of pot marigold (*Calendula officinalis* L.) under different combinations of L-arginine (L-Arg) and seaweed extract (SW) treatments.

| L-Arginine  (mg L^-1^) | Seaweed  (g L^-1^) | FN | FD  (mm) | FL  (days) | RDM  (%) | SDM  (%) | EL  (%) | RWC  (%) | P_n_  (µmol CO_2_ m^-2^ s^-1^) | E  (mmol H_2_O m^-2^ s^-1^) | g_s_  (mmol m^-2^ s^-1^) | C_i_  (µmol mol^-1^) | WUE  (µmol CO_2_ mmol^-1^ H_2_O) |
| --- | --- | --- | --- | --- | --- | --- | --- | --- | --- | --- | --- | --- | --- |
| 0 | 0 | 8.00 ± 0.59 e | 52.91 ± 1.49 d | 8.42± 0.08 e | 20.23± 1.60 de | 12.38± 0.12 a | 83.01± 1.46 a | 57.92 ± 0.24 c | 2.08± 0.49 a | 3.44 ± 0.12 a | 0.15± 0.00 a | 167.42± 4.16 a | 0.60 ± 0.13 a |
|  | 1 | 12.33 ± 0.90 cd | 61.67 ± 0.00 c | 9.50± 0.26 d | 26.89± 1.82 b | 13.04± 0.77 a | 71.93± 3.10 b | 58.02± 0.99 c | 2.27± 0.12 a | 3.37± 0.18 a | 0.12± 0.01 a | 150.57± 5.42 b | 0.67 ± 0.07 a |
|  | 2 | 13.00 ± 0.59 bc | 64.38± 1.06 c | 11.81± 0.50 ab | 22.40± 0.50 c-e | 14.13± 0.38 a | 66.87± 10.11 d | 63.31± 0.45 b | 2.65±0.11 a | 2.57±0.19 a | 0.10±0.00 a | 145.42± 5.14 b | 1.30 ± 0.12 a |
| 150 | 0 | 11.00 ± 0.59 d | 60.70±0.29 c | 9.55± 0.24 d | 18.63± 0.71 e | 11.91± 0.12 a | 72.95± 1.41 b | 63.05± 1.44 b | 3.42± 0.22 a | 3.08±0.20 a | 0.13±0.00 a | 102.42± 1.85 c | 1.11 ±0.12 a |
|  | 1 | 12.33 ± 0.34 cd | 63.01± 2.04 c | 10.97± 0.47 bc | 20.55± 0.37 de | 13.03± 0.26 a | 68.09± 1.33 bc | 63.47± 0.59 b | 4.02±0.27 a | 2.99±0.20 a | 0.12±0.00 a | 113.72± 6.27 c | 1.35±0.18 a |
|  | 2 | 13.33 ± 0.34 bc | 65.05± 2.25 c | 11.06± 0.41 bc | 35.08± 1.27 a | 14.44± 0.28 a | 57.04± 1.81 d | 61.29± 1.08 b | 5.05±0.38 a | 2.23±0.22 a | 0.09±0.01 a | 101.54± 5.13 c | 2.26± 0.30 a |
| 300 | 0 | 13.33± 0.34 bc | 76.76± 1.10 b | 10.36± 0.27 cd | 21.51± 1.28 de | 13.51± 0.14 a | 71.05± 0.92 b | 62.95± 1.24 b | 6.90±0.01 a | 2.97± 0.09 a | 0.10±0.01 a | 63.27± 1.13 d | 2.32±0.07 a |
|  | 1 | 14.33± 0.34 ab | 76.49± 1.70 b | 12.01± 0.27 ab | 23.38± 1.50 b-d | 14.54± 0.04 a | 63.26± 1.13 c | 61.29± 1.12 b | 7.39±0.19 a | 2.59±0.20 a | 0.09±0.01 a | 61.61±5.11 d | 2.86±0.30 a |
|  | 2 | 15.00± 0.59 a | 88.35± 1.89 a | 12.81± 0.28 a | 25.61± 0.71 bc | 15.44± 0.24 a | 54.30± 1.55 d | 66.43± 0.81 a | 8.13±0.20 a | 2.29±0.07 a | 0.07±0.00 a | 59.12±3.27 d | 3.53±0.16 a |

FN: flower number; FD: Flower diameter; FL: Flower longevity; RDM: Root dry matter; SDM: Shoot dry matter; EL: Electrolyte leakage; RWC: Relative water content; Pn: Net photosynthetic rate; E: Transpiration rate; gs: Stomatal conductance; Ci: Intercellular CO₂ concentration; WUE: Water use efficiency.

**Supplementary Table S2.** Mean values (± standard error) for chlorophyll fluorescence characteristics, photosynthetic pigment contents and biochemical parameters of pot marigold (*Calendula officinalis* L.) under different combinations of L-arginine (L-Arg) and seaweed extract (SW) treatments.

| L-Arginine  (mg L^-1^) | Seaweed  (g L^-1^) | F0 | Fv | Fm | Fv/Fm | NPQ | Chl a  (mg g^-1^ FW) | Chl b  (mg g^-1^ FW) | Chl T  (mg g^-1^ FW) | Car  (mg g^-1^ FW) | CAT  (U mg^-1^ protein) | POD  (U mg^-1^ protein) | TSP  (µg g^-1^ FW) | PC  (µmol g^-1^ FW) |
| --- | --- | --- | --- | --- | --- | --- | --- | --- | --- | --- | --- | --- | --- | --- |
| 0 | 0 | 84.50 ± 0.29 a | 180.17±8.34 c | 264.67±8.57 b | 0.68±0.01 d | 0.72±0.03 a | 0.93±0.01 f | 0.35±0.01 e | 1.43±0.03 d | 0.23±0.00 f | 0.76±0.01 e | 0.07±0.01 a | 157.81±3.40 e | 1.97±0.14 a |
|  | 1 | 73.33± 0.90 de | 202.00±2.70 b | 275.33±3.24 b | 0.73±0.00 c | 0.58±0.01 a | 1.11±0.03 cd | 0.47±0.00 bc | 1.73±0.04 b | 0.29±0.00 e | 0.82±0.03 de | 0.12±0.01 a | 160.51±3.07 de | 2.01±0.02 a |
|  | 2 | 71.00±0.59 e | 224.00±11.02 a | 295.00±10.60 a | 0.76±0.01 b | 0.45±0.02 a | 1.11±0.03 cd | 0.49±0.01 b | 1.73±0.06 b | 0.30±0.01 de | 0.94±0.03 d | 0.15±0.01 a | 164.34±2.15 de | 2.14±0.01 a |
| 150 | 0 | 82.33±1.48 a | 228.33±5.07 a | 310.67±4.13 a | 0.73±0.01 c | 0.59±0.02 a | 1.02±0.01 e | 0.38±0.02 de | 1.50±0.01 cd | 0.30±0.01 de | 0.90±0.04 d | 0.11±0.01 a | 175.48±2.68 c-e | 1.98±0.04 a |
|  | 1 | 78.00±0.59 b | 226.33±7.35 a | 304.33±7.31 a | 0.74±0.01 bc | 0.42±0.00 a | 1.09±0.01 d | 0.41±0.01 cd | 1.64±0.02 bc | 0.34±0.01 c | 1.24±0.06 c | 0.17±0.01 a | 177.02±6.77 cd | 2.13±0.05 a |
|  | 2 | 74.67±1.22 cd | 228.67±1.70 a | 303.33±1.22 a | 0.76±0.01 b | 0.38±0.01 a | 1.19±0.00 ab | 0.58±0.01 a | 1.95±0.01 a | 0.36±0.01 b | 1.36±0.05 b | 0.21±0.02 a | 183.82±2.87 c | 2.20±0.08 a |
| 300 | 0 | 76.33±1.48 bc | 229.33±3.02 a | 305.67±1.70 a | 0.75±0.01 bc | 0.46±0.02 a | 1.16±0.02 bc | 0.47±0.05 bc | 1.99±0.07 a | 0.31±0.01 d | 1.23±0.08 c | 0.20±0.01 a | 172.02±6.29 c-e | 2.27±0.18 a |
|  | 1 | 73.00±0.59 de | 237.00±1.18 a | 310.00±0.59 a | 0.76±0.00 b | 0.33±0.02 a | 1.19±0.02 ab | 0.50±0.02 b | 1.66±0.11 b | 0.35±0.00 b | 1.41±0.02 ab | 0.22±0.01 a | 216.20±9.48 b | 2.46±0.20 a |
|  | 2 | 68.00±0.59 f | 242.67±2.07 a | 310.67±2.38 a | 0.79±0.00 a | 0.23±0.01 a | 1.24±0.01 a | 0.57±0.01 a | 1.75±0.02 b | 0.40±0.01 a | 1.51±0.02 a | 0.27±0.01 a | 244.60±7.91 a | 2.55±0.18 a |

F_0_: minimum fluorescence; F_v_: variable fluorescence, F_m_: maximum fluorescence; F_v_/F_m_: maximum quantum yield of PSII; NPQ: Non-photochemical quenching; Chl a: chlorophyll a; Chl b: chlorophyll b; Chl T: total chlorophyll; Car: total carotenoids; CAT: catalase activity; POD: peroxidase activity; TSP: total soluble protein; PC: proline content.
